# Pollinators enhance crop yield and shorten the growing season by modulating plant functional characteristics: A comparison of 23 canola varieties

**DOI:** 10.1038/s41598-019-50811-y

**Published:** 2019-10-02

**Authors:** George C. Adamidis, Ralph V. Cartar, Andony P. Melathopoulos, Stephen F. Pernal, Shelley E. Hoover

**Affiliations:** 1 Department of Biological Sciences, University of Calgary, Calgary, Canada; 2 Department of Horticulture, Oregon State University, Corvallis, OR USA; 3 Agriculture and Agri-Food Canada, Beaverlodge Research Farm, Beaverlodge, AB Canada; 4 Alberta Agriculture and Forestry, Lethbridge, AB Canada

**Keywords:** Agroecology, Ecophysiology

## Abstract

Insect pollination of flowers should change the within-season allocation of resources in plants. But the nature of this life-history response, particularly regarding allocation to roots, photosynthetic structures, and flowers, is empirically unresolved. This study uses a greenhouse experiment to investigate the effect of insect pollination on the reproductive output of 23 varieties of a globally important crop—canola (*Brassica napus*). Overall, insect pollination modified the functional characteristics (flower timing & effort, plant size & shape, seed packaging, root biomass) of canola, increasing seed production and quality, and pollinator dependence. Reproductive output and pollinator dependence were defined by strong trait trade-offs, which ranged from more pollinator-dependent plants favouring early reproductive effort, to less pollinator-dependent plants favouring a prolonged phenology with smaller plant size and lower seed quality. Seed production decreased with pollinator dependence in the absence of pollinators. The agricultural preference for hybrid varieties will increase seed production compared to open-pollinated varieties, but, even so, pollinators typically enhance seed production of both types. Our study elucidates how insect pollination alters the character and function of a globally important crop, supporting optimization of yield via intensification of insect pollination, and highlights the beneficial effects of insect pollination early in the season.

## Introduction

The allocation of resources between growth and reproduction is a central theme in life history evolution^1^, directly affecting age and size at maturity^2^. In the case of plant life histories, understanding this tradeoff is potentially important for global crop production. Plants, due to their sessile nature and constraints on dispersal, have evolved a variety of strategies that allow them to optimize fitness across a range of biotic and abiotic environments. Phenotypic plasticity - i.e., the ability of a genotype to express alternative phenotypes depending on the environment, is considered the primary such strategy to cope with environmental heterogeneity^3^, and is common in many plant traits (reviewed by Dudley *et al*.^4^). Plants respond adaptively to environmental change by altering traits related to biomass allocation, morphology, physiology, architecture, and phenology^5–8^, resulting in inter- and intra-specific plant trait variation (e.g.,^9^). Trait evolution involves allocation of limited resources between alternative functions^10^, often mediated by responses to stress^11^. To date, studies addressing the factors that determine adaptive plant responses have mostly considered the effect of the abiotic environment (e.g.,^8,12^), overlooking the important role of biotic interactions on phenotypic plasticity (reviewed by Valladares *et al*.^11^).

Insect pollination is one such biotic interaction that might strongly determine plasticity of trait variation in plants. For example, by investing in flowers, plants might have fewer resources to invest in seeds^13^. Similarly, receipt of high-quality pollen early in a perennial plant’s life history might favour greater early reproductive effort, with the consequence of lower later reproductive effort, as inferred from studies of pollen limitation^14^. In general, we expect pollination to have profound consequences on costs of reproduction in plants^15,16^. Because 75% of seed and fruit production of the major global field crops relies, at least to some extent, upon insect pollination^17^, it is important to understand the effects of insect pollination on resource allocation and plastic trait responses in crop plants.

Canola (*Brassica napus L*.), also known as oilseed rape or rapeseed, is one of the most important crops worldwide^18,19^. While canola is capable of self-pollination, its floral physiology (i.e., bilaterally symmetrical yellow flowers with sticky pollen) implies insect rather than wind pollen dispersion^20^. However, while benefits of insect visitation in canola seed production and quality have reported (e.g.^21–25^), some studies present inconsistent results (e.g.^26^). Differences among canola varieties (e.g.^27^) could explain these results regarding the effect of insect pollination on canola seed production, meriting a more thorough examination of pollinator dependence (i.e. the extend that a canola plant/variety depends on pollinator visitation for fruit set) and its associated traits among canola varieties.

For almost 60 years, canola has been the focus of breeding programs, intensively producing new certified varieties^28^. Depending on the breeding system used in the parental generation, canola varieties are classified either as open-pollinated (OP) or hybrid (H). Open-pollinated canola varieties are developed by selecting and crossing the most efficient genotypes^29^, while hybrid varieties result from selecting and mating two inbred lines, crossed so as to maximize heterosis. Hybrid varieties have come to dominate the global market^30,31^, but some of their important characteristics, such as responses to pollinators, are unreported^32^. For example, has the transition from use of open-pollinated to use of hybrid varieties resulted in a change in pollinator dependence? However, considering the economic importance of canola crops globally, the global decline in wild pollinators^33^, and the fact that human food security decreases with increased crop pollinator dependence^34^, the importance of examining the traits and pollination dependencies of canola becomes apparent.

To determine how insect pollination affects reproductive output and pollinator dependence of a globally important crop, as mediated by key vegetative and phenological traits, we experimentally evaluated the functional output to insect visitation of 23 commercially available canola varieties, comprising 8 open-pollinated and 15 hybrid varieties. Specifically, the following four questions were addressed: (1) Do different canola varieties and variety types (OP & H) alter their functional characteristics in response to insect pollination? (2) Does insect pollination increase seed production and quality across canola varieties and variety types, and if so, through which specific vegetative and phenological trait alterations is this achieved? (3) Does pollinator dependence vary across canola varieties and variety types, and, if so, which traits characterize a ‘pollinator-dependent’ canola plant? And (4) are pollinator-dependent canola varieties/variety types more, or less, productive? We addressed these questions in a greenhouse experiment.

## Results

### Functional character

The overall functional character of canola (i.e. multivariate sets of functional characteristics/traits (i.e., “syndromes”) related to flower timing, flower effort, plant size & shape, seed packaging and root biomass, MANOVA reported in Supplementary Table S1) was affected by pollinators, canola type, and variety. Canola varieties differed in the response of their functional trait syndromes to pollinators (see Pollinators × Variety interaction in Supplementary Table S1). Pollinator-associated trait changes were typical, as 17 out of the 23 varieties (5/8 of open-pollinated and 12/15 of hybrid canola varieties) altered their functional traits in response to pollination (multivariate pairwise comparisons; *P* < 0.05). Given the multivariate responses we detected, we next examined the univariate main effects, to see how each functional trait syndrome differed according to pollinator treatment.

Each canola functional trait syndrome and root biomass differed between pollinator treatments (*P* < 0.001 in all cases; Supplementary Table S2). Canola plants exposed to pollinators allocated fewer resources to flower production (i.e., flowering effort), to the production of pods (i.e., seed packaging) and to both above- and below-ground plant growth (plant size & shape and root biomass, respectively), while flowering earlier than plants in the no-pollinator treatment (Supplementary Fig. S1). Although the variety types (OP vs. H) did not differ in flower production, seed packaging, or root biomass (*P* > 0.05), variety types did differ in two other functional traits: the open-pollinated varieties allocated more resources to above ground plant growth (*P* < 0.003) and flowered later (*P* < 0.001) relative to hybrid varieties (Supplementary Table S2; Supplementary Fig. S1). Canola varieties also presented distinct functional trait syndromes (*P* < 0.05 in all traits; Supplementary Table S2). Finally, differences in functional traits between canola variety types were unaltered by pollinators (Pollination × Variety type interaction, Supplementary Table S2; Supplementary Fig. S1), while the effects of pollination on flower timing, flowering effort and plant size & shape differed among canola varieties (Pollination × Variety’ interaction term, Supplementary Table S2). Hence, all plant functional traits differed among varieties and pollination treatments, but, other than flowering effort, interactions between pollination and variety were either modest or non-significant.

### Reproductive output

**Figure 1:**
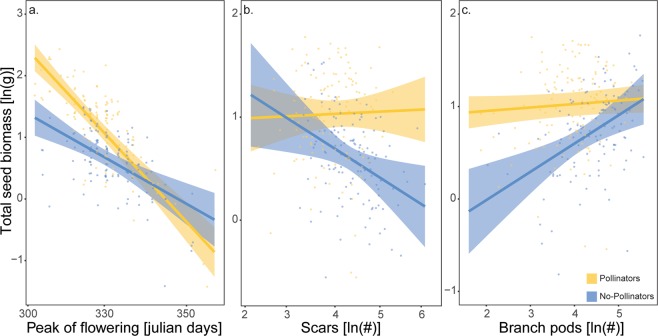
Partial residuals, prediction lines and confidence bands showing interactive effects of canola functional traits (**a**) -(Peak_of_flowering)^−2^; (**b**) ln (Number of scars) and (**c**) ln (Number of branch pods)) and pollination treatment (pollinators in yellow, no-pollinators in blue) on ln-transformed Total seed biomass. Back-transformed values of peak of flowering are presented on the x-axis of (**a**).

Total canola seed biomass was, on average, higher in plants exposed to insect pollination (*F*_1,232_ = 8.96, *P* = 0.003; Supplementary Table S3), with canola varieties also differing in their seed biomass (*F*_1,232_ = 4.45, *P* = 0.036; Supplementary Table S3), and with hybrid variety type producing the highest seed biomass (*F*_1,232_ = 15.90, *P* < 0.001; Supplementary Table S3; Supplementary Fig. S2). On average, canola seed biomass decreased with a later peak of flowering (*F*_1,232_ = 120.11, *P* < 0.001; Supplementary Table S3; Supplementary Fig. S3), and increased with increasing root biomass (*F*_1,232_ = 36.76, *P* < 0.001; Supplementary Table S3; Supplementary Fig. S3), plant height (*F*_1,232_ = 37.31, *P* < 0.001; Supplementary Table S3; Supplementary Fig. S3), number of primary branches (*F*_1,232_ = 5.26, *P* = 0.023; Supplementary Table S3; Supplementary Fig. S3), and number of main stem pods (*F*_1,232_ = 7.25, *P* = 0.007; Supplementary Table S3; Supplementary Fig. S3). Pollinator visitation exacerbated the negative relationship between seed production and time of peak flowering, resulting in higher total seed biomass in canola plants with an earlier peak of flowering (Pollination × Peak of flowering interaction: *F*_1,232_ = 11.78, *P* < 0.001; Supplementary Table S3; Fig. 1a). Furthermore, the presence of pollinators removed both the negative effect of number of scars (Pollination × #Scars interaction: *F*_1,232_ = 5.59, *P* = 0.019; Supplementary Table S3; Supplementary Fig. 1b) and the positive effect of number of branch pods (Pollination × #Branch pods interaction: *F*_1,232_ = 8.90, *P* = 0.003; Supplementary Table S3; Supplementary Fig. 1c) on total seed biomass, always resulting in higher or equivalent seed production in comparison to the plants grown in the absence of pollinators. Overall, bigger, earlier-flowering plants with more investment in main stems (not secondary branches) had the highest seed production. But some of these simple responses depended on pollinator presence. Pollinators increased the value of earlier phenology, and removed the relationship between scars or branch pods and total seed biomass (in both cases keeping seed production at its highest in the presence of pollinators).

**Figure 2:**
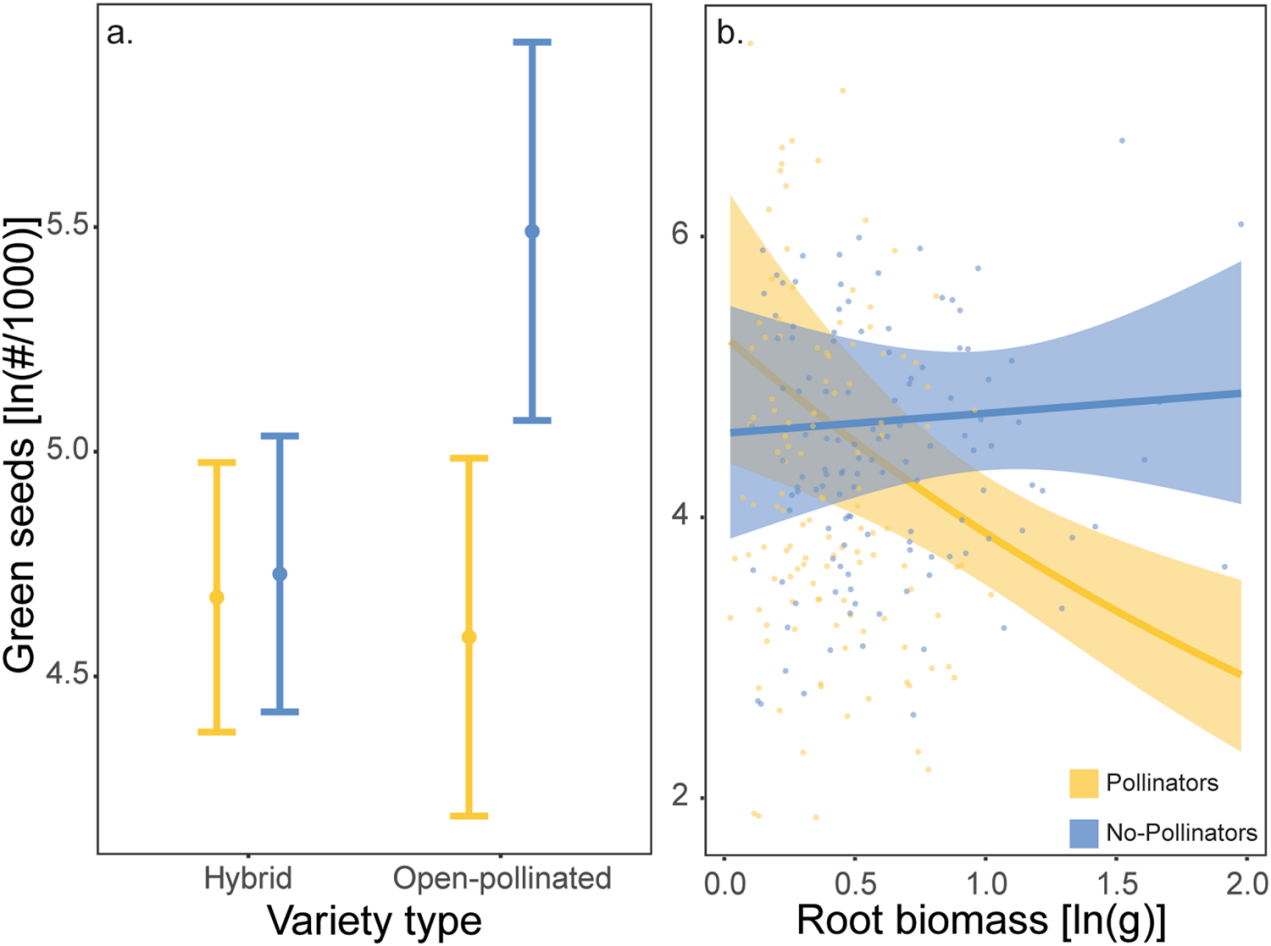
(**a**) Model estimated mean (ln-transformed) number of green seeds per 1000 seeds for hybrid and open-pollinated canola varieties in the presence (in yellow) and absence (in blue) of bumble bees. Error bars 95% confidence intervals. (**b**) Partial residuals, prediction lines and confidence bands showing interactive effects of canola root biomass (ln-transformed) and pollination treatment (pollinators in yellow, no-pollinators in blue) on ln-transformed number of green seeds per 1000 seeds.

Green seeds are under-developed, and therefore represent a fitness and economic cost. There was a marginal tendency (*P* = 0.071; Supplementary Table S2) for canola plants not visited by pollinators to produce more green seeds, while variety type (OP versus H) and variety, on average, did not differ in their green seed count (*P* > 0.05 in all cases; Supplementary Table S2). Green seeds were more common in canola plants with a later peak of flowering (*Z* = 6.32, *P* < 0.001; Supplementary Table S4; Supplementary Fig. S4), but were reduced with increasing number of flowers at the peak of flowering (*Z* = −2.66, *P* = 0.008; Supplementary Table S4; Supplementary Fig. S4). The number of green seeds was also lower for tall plants (*Z* = −2.38, *P* = 0.017; Supplementary Table S4; Supplementary Fig. S4), and plants with high root biomass (*Z* = −2.07, *P* = 0.038; Supplementary Table S4; Supplementary Fig. S4). On average, fast-growing and fast-maturing canola plants, with the ability to produce high numbers of flowers at the peak of flowering, produced fewer green seeds and thus higher quality seeds. Insect pollination decreased the number of green seeds for open-pollinated canola varieties, but not for hybrid varieties, which had low numbers of green seeds in both pollination treatments (Pollination × Variety type interaction: *Z* = 2.73, *P* = 0.006; Supplementary Table S4; Fig. 2a). The presence of pollinators interacted with root biomass to affect the number of green seeds: in the presence of pollinators, plants with larger roots produced more green seeds but in the absence of pollinators, root biomass was unrelated to green seed production (Pollination × Root biomass interaction: *Z* = 2.08, *P* = 0.037; Supplementary Table S4; Supplementary Fig. 2b). Overall, green seeds increased with later peak flowering, and decreased with plant size (i.e., root biomass, plant height) and number of flowers, but lack of bees removed the effect of roots on green seeds.

### Pollinator dependence

**Figure 3:**
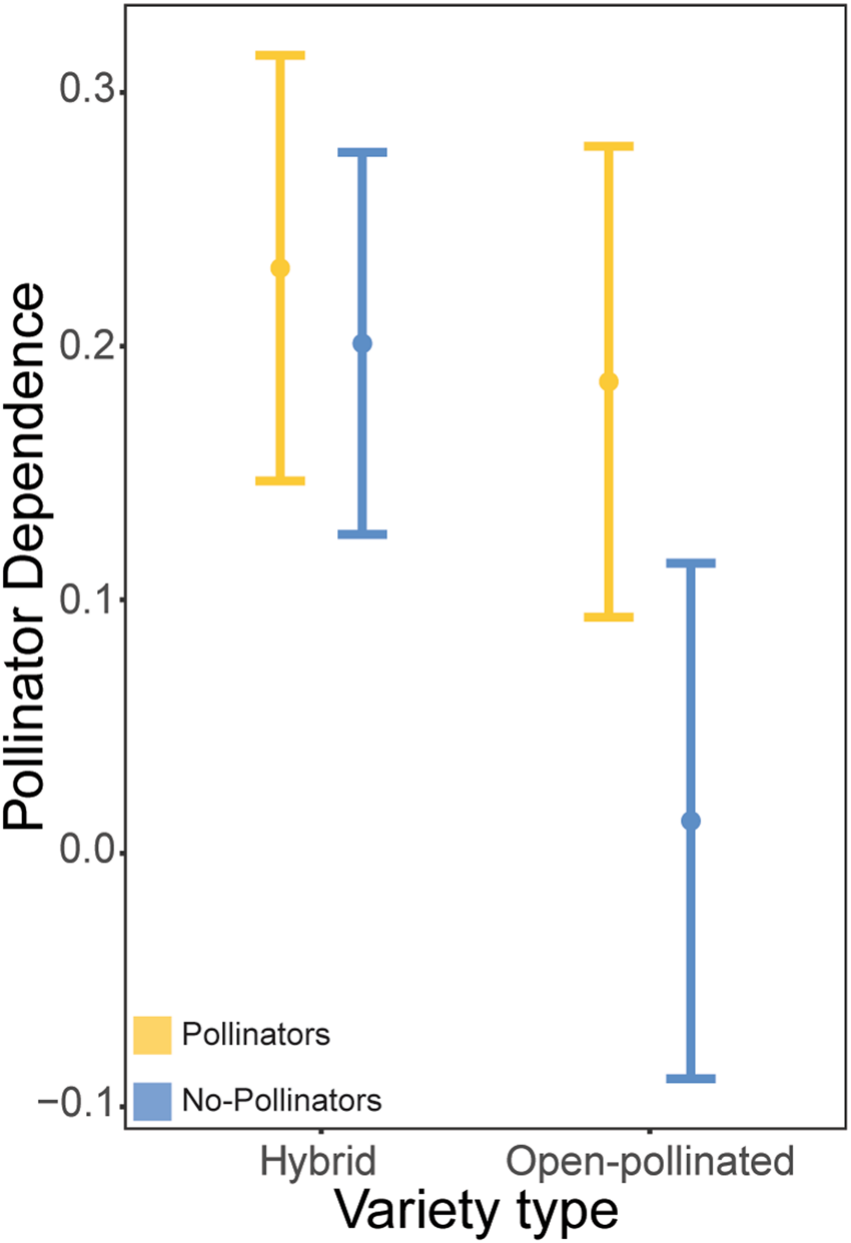
Model estimated mean pollinator dependence for hybrid and open-pollinated canola varieties in the presence (in yellow) and absence (in blue) of bumble bees. Error bars 95% confidence intervals.

Pollinator dependence was, on average, higher in canola plants exposed to insect pollination (*F*_1,231_ = 118.21, *P* < 0.001; Supplementary Table S5). Although canola varieties differed in their dependence on pollinators (*F*_1,231_ = 8.46, *P* = 0.004; Supplementary Table S5), the two types of canola (OP versus H), on average, showed similar pollinator dependence (*P* > 0.05; Supplementary Table S5). Dependence on insect pollination decreased, on average, in plants with a later peak of flowering (*F*_1,231_ = 66.18, *P* < 0.001; Supplementary Table S5; Supplementary Fig. S5). The most pollinator-dependent canola plants produced more flowers at the peak of flowering (*F*_1,231_ = 9.69, *P* = 0.002; Supplementary Table S5; Supplementary Fig. S5), had larger roots (*F*_1,231_ = 4.07, *P* = 0.045; Supplementary Table S5; Supplementary Fig. S5), were taller (*F*_1,232_ = 6.22, *P* = 0.013; Supplementary Table S5; Supplementary Fig. S5), and produced more main stem pods (*F*_1,231_ = 4.79, *P* = 0.029; Supplementary Table S5; Supplementary Fig. S5), while there was a marginal tendency of canola plants that produced higher numbers of branch pods to also be more pollinator-dependent (*P* = 0.076; Supplementary Table S5). The presence of pollinators increased pollinator dependence of canola plants, especially for open-pollinated varieties (Pollination × Variety type interaction: *F*_1,231_* = *4.67, *P* = 0.032; Supplementary Table S5; Fig. 3). Although our results demonstrate a small increase in pollinator dependence of the hybrid varieties in the presence of pollinators, on average hybrid varieties were highly pollinator dependent in both presence and absence of pollinators. Pollinator visitation reversed the positive relationship between pollinator dependence and peak of flowering, rendering the canola plants with an earlier peak of flowering more pollinator dependent (Pollination × Peak of flowering interaction: *F*_1,231_ = 113.33, *P* < 0.001; Supplementary Table S5; Supplementary Fig. S6). Furthermore, the presence of pollinators accentuated the positive effect of number of flowers at peak on pollinator dependence (*P* = 0.052; Supplementary Table S5). Finally, insect pollination generated positive effects on pollinator dependence of root biomass (Pollination × Root biomass interaction: *F*_1,231_ = 16.14, *P* < 0.001; Supplementary Table S5; Supplementary Fig. S6), plant height (Pollination × Plant height interaction: *F*_1,231_ = 13.69, *P* < 0.001; Supplementary Table S5; Supplementary Fig. S6), number of main stem pods (Pollination × #Main stem pods interaction: *F*_1,231_ = 14.84, *P* < 0.001; Supplementary Table S5; Supplementary Fig. S6) and number of branch pods (Pollination × #Branch pods interaction: *F*_1,231_ = 6.13, *P* = 0.014; Supplementary Table S5; Supplementary Fig. S6).

### Seed production and pollinator dependence

**Figure 4:**
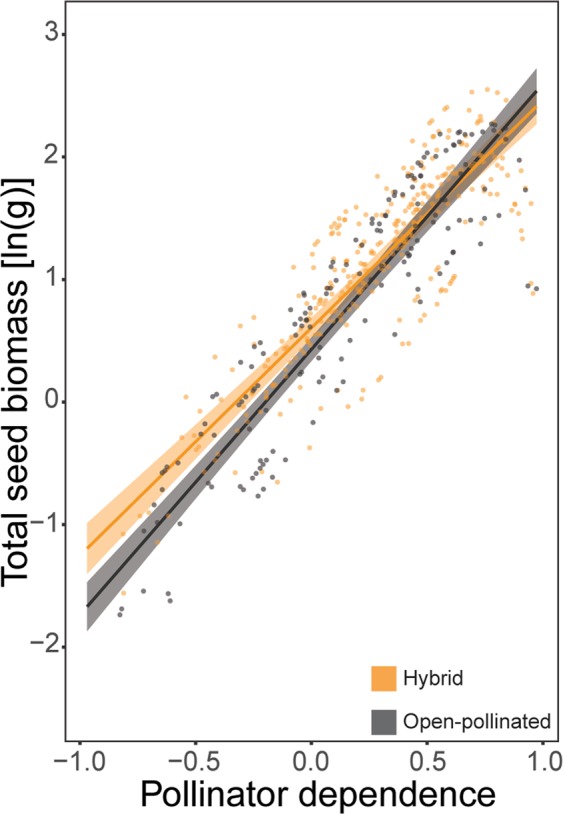
Partial residuals, prediction lines and confidence bands showing interactive effects of pollinator dependence and canola variety type (hybrid in orange, open-pollinated in grey) on ln-transformed total seed biomass.

**Figure 5:**
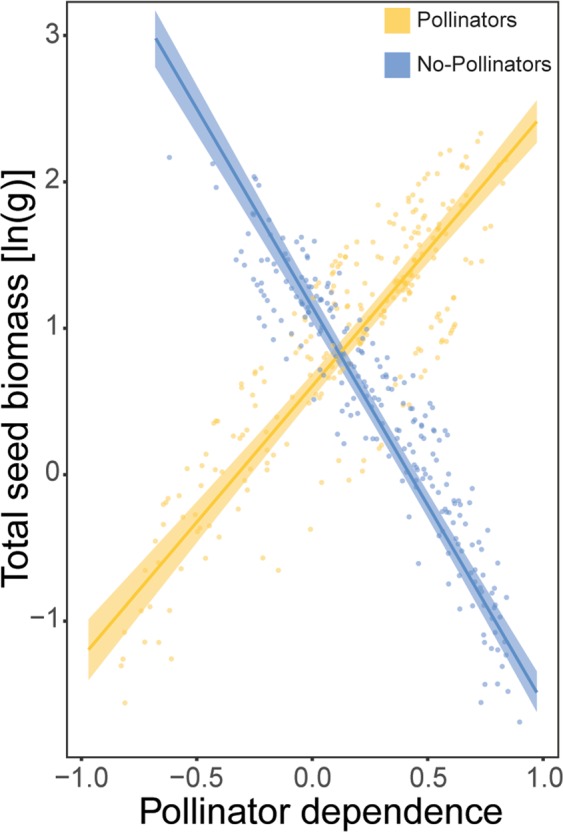
Partial residuals, prediction lines and confidence bands showing interactive effects of pollinator dependence and pollination treatment (pollinators in yellow, no-pollinators in blue) on ln-transformed total seed biomass.

Total seed biomass was, on average, higher on pollinator dependent canola plants (*F*_1,470_ = 499.78, *P* < 0.001; Supplementary Table S6). Pollinator dependence had a stronger effect on total seed biomass than did the effects of pollination treatment (*F*_1,470_ = 124.54, *P* < 0.001; Supplementary Table S6), variety type (*F*_1,470_ = 60.53, *P* < 0.001; Supplementary Table S6) and variety (*F*_1,470_ = 22.79, *P* < 0.001; Supplementary Table S6). Seed production was enhanced by increasing pollinator dependence, and this effect was strongest for the open-pollinated varieties (Pollinator dependence × Variety type interaction: *F*_1,470_ = 9.19, *P* = 0.003; Supplementary Table S6; Fig. 4). Finally, the effect of pollinator dependence on seed production differed profoundly between pollination treatments: positive in the presence of pollinators, and negative in the absence of pollinators (Pollinator dependence × Pollination interaction: *F*_1,470_ = 1935.87, *P* < 0.001; Supplementary Table S6; Fig. 5).

## Discussion

We found that functional traits of canola varied across varieties and variety types, but pollinator visitation was the most important factor determining the overall functional character of canola (see *F*-values in Supplementary Table S1). The transition in use from open-pollinated to hybrid canola varieties, representing the evolution of the canola seed production industry and breeding programs, is associated with a shift towards smaller and earlier-flowering canola plants. In a similar sense, González-Paleo & Ravetta^35^ showed that traits associated with stability of seed yield were altered during artificial selection for increased seed production, with plants in selected lines exhibiting traits that differed from their wild counterparts, particularly in reducing early vigour and growth rate. In our study, the effect of pollinators on the overall functional character of canola was similar for open-pollinated and hybrid varieties, but different for individual canola varieties, meriting a variety-level focus to understand inconsistencies of insect pollination benefits on canola seed production in past research (e.g.^36^).

In particular, we found that canola plants exposed to pollinators allocated fewer resources to flower production (i.e., flowering effort), to the production of pods (i.e., seed packaging) and to both above- and below-ground plant growth (plant size & shape and root biomass, respectively), while flowering earlier than plants in the no-pollinator treatment (Supplementary Fig. S1). These results, are consistent with studies demonstrating that insect-pollinated canola flowers are shorter-lived^37^, that pollinator visitation decreases the number of produced flowers by canola plants^38^ and, consequently, that canola plants visited by pollinators have shorter flowering periods^39,40^.

In the absence of pollinators, canola plants continue to bloom – through the production of additional flowers on new branches^41^ – until they have used up acquired resources^38,42^. A similar temporal win-stay/lose-shift pattern is observed in the life histories of many wild plants, and is considered an adaptation to uncertainty in seed formation and development (e.g., inconsistent pollination, fluctuating resource availability, flower and pod damage^43–45^). In annual plants, early and continuing reproduction is also favoured over bang-bang reproduction (growing to an optimal size before switching to reproduction) when the timing of the season end (and therefore plant lifespan) is unpredictable^46–48^. In contrast, when seed predation is higher later in the season, early reproduction is optimal^49^. In temperate agro-ecosystems, it is likely that canola plants do indeed face weather-related uncertainty in timing of season end. Such uncertainty in season end, rather than greater seed predation later in the season, is the most plausible influence favouring early reproduction. Consistent with this flowering strategy, we found that pollinator visitation reduces uncertainty in seasonal reproduction by setting seed early, allowing canola plants to fully realize their somatic investment in the ovules of early flowers, and thereby avoid the costs of producing late flowers and associated compensatory above- and below-ground “infrastructure” (i.e., branches and root tissue). Although pollinators are often managed for their crop-pollination services and thus pollination is widely considered as an agronomic input (e.g.^50–52^), our results show that pollinator visitation affects the canola functional characteristics in a distinct way relative to the effect of other agronomic inputs such as fertilizer or water. More specifically, several studies show that the addition of agronomic inputs such as fertilization or irrigation increase plant height, number of branches, number of pods and root biomass (e.g.^53–55^) of canola plants. In contrast, we found that bumble bee pollination decreases the resources spent by canola plants in future reproduction by decreasing the days to maturity. In other words, in response to our first question, canola plants visited by pollinators alter their functional characteristics by allocating resources in a way that enables them to reach their maximum capacity faster.

The present study shows that pollinator visitation, on average, increased canola seed production. However the degree of benefit derived from insect pollination depended on both variety and variety type. Many studies have previously confirmed that insect pollination increases canola yield (e.g.^21,22,25,56^), but there are inconsistencies in the magnitude of pollination benefits (46% yield increase^21^, 18% yield increase^22^, no yield increase^26^). Our results demonstrate that these differences may be partly explained by varietal differences, varying from 23.7% decrease to 345.7% increase of seed production (mean = 94.3% increase). Hudewenz *et al*.^24^ found that differences in yield responses to insect pollination of four rapeseed varieties were related to variety-specific pollinator dependence, and, as we discuss below, there is a high inter-varietal variability in canola pollinator dependence. In addition, in common with previous studies^57^, hybrid canola varieties always had higher seed production than open-pollinated ones. This is expected because hybrid varieties gain a reproductive output advantage from heterosis^30,58^, a transient state in nature. Canola plants that had higher seed production were, on average, taller, with larger roots, produced more main stem branches with more pods, and had an earlier peak of flowering. These functional characteristics are associated with a more acquisitive resource use strategy, favoured in resource-rich environments like agroecosystems, where selective breeding for yield may shift crop functional traits toward the resource acquisitive side of any trait economic spectra^59,60^.

We found that pollinator visitation increases canola seed production by advancing flower phenology (i.e., an earlier peak of flowering), and subsequently decreasing investment in secondary branches. Although previous studies have reported pollination effects on phenological and vegetative canola traits (e.g.^38–40,61^), this is the first time that the precise functional traits through which insect pollination affects canola seed production are revealed (Fig. 1). Similar efforts to identify sets of functional traits that influence phenological, reproductive and photosynthetic responses to global warming have been undertaken in wild plants^62,63^. Such studies emphasize the importance of considering specific functional traits to understand how different species will be affected by global environmental change. In the same sense, shedding light on the ability of insect pollinators to modify key functional traits of globally important crops is important for ensuring the security of the future global food supply, especially under ongoing global decline in wild and managed pollinators^33^.

Apart from the positive effect of pollinators on canola seed production, we found a tendency for canola plants visited by pollinators to also increase their seed quality by decreasing the proportion of green seed. By the same token, sufficient insect pollination increases crop quality not only in canola^23,64^ but also in several other fruits and vegetables (e.g. apples^65^, almonds^66^, pears^67^, passion fruit, tomatoes and capsicum^68^, strawberries^69^, blueberries^70^). Although, on average, different canola varieties and variety types produced similar amounts of late maturing seeds, the production of green seeds was lower in taller canola plants with larger roots, who had an earlier peak of flowering and produced more flowers at the peak of flowering. Hence, canola plants that produced higher-quality seeds had a set of traits enabling them to acquire and use resources rapidly (i.e., tall, with large roots), in a way that not only produced phenological advances (i.e., earlier peak of flowering), but also promoted a higher number of flowers at the peak of flowering. Interestingly, canola plants that produced high quantity and quality seeds shared almost identical functional syndromes: both resorted to more resource-acquisitive strategies with earlier flowering. The only difference in these functional syndromes is that highly productive canola plants additionally prioritized main stem structural and reproductive effort, while canola plants with high-quality seed production additionally had more synchronous and massive flower production at the peak of flowering. Although seed quality and quantity can be traded-off at high fruit loads (e.g.^71^), this is not the case in crops of indeterminate flowering such as canola crops^22^. We found that seed quality of open-pollinated canola varieties can substantially benefit from pollinator visitation, but not so for hybrid varieties, whose low green seed numbers were unrelated to the presence of pollinators.

Bee visitation increased canola seed quality by increasing the value of investment in below-ground plant growth. Different abiotic/biotic factors controlling flowering phenology, and consequently the uniformity of seed emergence, are known to be perceived by different plant organs^72^, with roots being the main organ participating in the perception of most of these factors (e.g., water & mineral availability and neighboring plant species^73^). In our study, canola plants who responded to pollinator visitation by either increasing or maintaining resources in roots produced higher quality seeds, by producing fewer late maturing “green” seeds. According to a well-documented trade-off between below- and above-ground resource acquisition also known as “optimal partitioning”, “balanced growth” or “functional equilibrium“^74–76^, plants preferentially allocate biomass to the organs with the highest demand for resources, and shift this allocation in response to changing conditions^77,78^. In support of the idea, tillage and nitrogen fertilization increased carbon allocation to the roots of a canola crop in a semi-arid dryland^79^. Similarly, although insect pollination, on average, decreased canola root size in our experiment, canola plants with adequate root investment to support rapid resource acquisition and use, eventually produced high-quality seeds by avoiding the potential costs of investment in late maturing seeds. Preventing uneven ripening in canola crops, with a concomitant increase in proportion of immature seeds at harvest, is economically important^80^. Immature (green) seed is associated with low quality canola oil^81^ and consequently with lower canola grading and reduced market value. Modifying a plant’s or crop’s functional character through insect pollination can affect its strategy of development, particularly its phenology or resource acquisition and use. As a result, shifts in plant traits at the individual level may affect ecosystem processes (e.g.^82–85^). In support of this argument, and in response to our second question, we found that pollinator visitation not only modified the functional character of canola, but also, through specific plastic responses, increased canola seed production and quality.

We found that canola varieties differed in their dependence on pollinator visitation for seed production. Similarly, Hudewenz *et al*.^24^ showed that differences in seed yield responses to pollination between four rapeseed varieties were related to inter-varietal differences in pollinator dependence. The high inter-varietal variability in canola pollinator dependence that we document suggests that variation in pollinator benefits may be large not only across crops (e.g.^67^) but also across varieties of the same crop, and that taking into consideration global averages of pollinator dependence at the species level, can be misleading^86^. In response to our third question, canola varieties used in this study that were more pollinator-dependent tended to be taller, with larger roots, more main stem pods, an earlier peak of flowering, and more flowers at the peak of flowering. On average, both pollinator dependence and canola seed production and quality are defined by similar trait trade-offs which run from more pollinator-dependent canola plants having high quantity and quality seed production with a potential for quick investment returns (larger plants with early peak of flowering and simultaneous/synchronized flower production at the peak of flowering) to less pollinator-dependent and less productive canola plants with a slower potential rate of return and opposite characteristics. We also highlight that in highly “plastic” crops such as canola^87–89^, the traits defining pollinator dependence can be affected by biotic factors such as pollinator visitation. In particular, taller canola plants with large roots, producing high numbers of main stems and with an earlier peak of flowering were more pollinator-dependent in the presence and less dependent in the absence of pollinators. Hence, canola plants growing in the presence of pollinators may possess traits that make them more dependent in pollinator visitation relative to canola plants growing in absence of pollinators (e.g., those in the middle of large fields).

Finally, we found that the relationship between seed production and pollinator dependence varied across pollination treatments and canola variety types. In particular, as expected, pollinator dependent varieties were more productive in the presence of pollinators, with the converse also true. In addition, at low pollinator dependence, hybrid varieties were more productive compared to open-pollinated ones, suggesting that hybrid canola may be more suitable when managed pollinators are not used, when availability of pollinators is uncertain, and/or in centers of large canola crops where wild pollinators may seldom reach. Thus, in response to our fourth question, pollinator dependent canola varieties are more productive in the presence of pollinators, but, at the same time, they typically fail to reach their maximum capacity without adequate pollinator visitation.

With 70% of agricultural crops being pollinator dependent^17^, and with the ongoing global decline in wild and managed pollinators^33^, there is a growing concern that the negative impact of crop pollinator dependence on global food production and stability^34^ will become even more pronounced. This study sheds light on how insect pollination alters the character and thus the function of a globally important crop: canola. The shifts in suites of canola traits that emerge from insect pollination can inform how canola crops will respond to changing environment (e.g., global environmental change, pollination declines), and on how they may influence ecosystem structure and function (according to the trait-based response-effect framework proposed by Lavorel and Garnier^90^). We found that insect pollination increases canola seed production and quality and pollinator dependence by altering the functional character of canola plants in a way that enables them to more quickly reach their maximum reproductive capacity. Considering that phenological delays substantially increase the risk of reproductive failure in canola crops (e.g.^91–93^), our results support the use of ecological intensification through insect pollination to optimize yield. Finally, we find high variability in pollinator dependence across canola varieties and variety types, highlighting the need for communication of canola pollination properties by the canola breeding companies to their growers. Selecting for increased seed yield may have significant effects on non-targeted canola traits, which consequently may affect processes and services at the ecosystem level.

## Methods

### Experimental design

To investigate the responses of canola to insect pollination, 23 past and present commercial canola varieties spanning the entire history of canola breeding (see Supplementary Fig. S7) and comprising eight open-pollinated (OP) and 15 hybrid (H) varieties, were grown under standardized conditions in two same-sized greenhouses at the Agriculture and Agri-Food Canada Lethbridge Research and Development Centre near Lethbridge, AB (49°38′N, 112°48′W). In September 2016, seeds of all varieties were sown in trays containing a standard potting soil and placed in a greenhouse maintained at a 16 h light diurnal cycle, with the period of light supplemented with 230 μmol m^−2^ s^−1^ illumination^94^, and at 22 °C/17 °C day/night temperature. After seven days, three seedlings of each variety were transplanted into one gallon pots of 15 cm diameter, and eleven pots per variety were placed in each of the two greenhouses (a total of 506 pots). In each greenhouse, pots were allocated in a 30 rows by 9 columns grid, with the rows arranged along a band of latitude. Pots were arranged according to a randomized complete block design, with 11 blocks being a combination of distance from outside edge of the grid, and latitude (Supplementary Fig. S7). Six edge pots from each variety and greenhouse (a total of 276 pots allocated in the outside 3 layers of the pot grid) were followed for measurement of phenological traits (see below). Phenology of the plants interior to these grid layers could not reliably be followed, so only their at-harvest traits were measured. To control for any potential greenhouse effect, the open-pollinated Westar variety was duplicated within each block and one of the two replicates was covered with a pollinator-excluding sleeve during the whole blooming period (see WES pots in black bold letters in Supplementary Fig. S7). In addition, 3 pots with mustard plants (*Brassica juncea*) and 3 pots with *Capsicum spp*. (used as biocontrol plants) were used to fill the center of the table (Supplementary Fig. S7). Pots were supplied with individual drip irrigation, which provided 100 ml of water twice a day for the duration of the experiment. As plants began to bolt, the terminal stem of the most central plant (hereafter focal plant) in each pot was tagged using a white plastic label. At 10% bloom, a commercial (Natupol, Koppert Ltd) bumble bee (*Bombus terrestris*) colony with 50–100 workers, was placed in one of the two greenhouses.

### Phenological traits

Flower production and phenology were scored daily in all edge focal plants (see above) throughout the blooming period. From these raw counts, the following phenological variables related to flowering were calculated: date of onset, peak, median and cessation, from which we calculated: duration of flowering, number of flowers produced at the flowering peak, and total flowers produced throughout the blooming period. All date variables were expressed as days since January 1^st^, while duration of flowering was estimated as the difference between onset and cessation of flowering.

### Vegetative traits

Beginning on the 10^th^ week after seeding, the lowest pod on the main stem on randomly selected non-focal plants from each variety was harvested and inspected for seed colour change. Plants were considered ripe when seeds in these oldest pods had completely changed colour from green to black. Once the fruiting was completed, and before the beginning of dehiscence of the ripe pods (between 22 December 2016 and 12 January 2017), all focal plants from the two greenhouses were harvested. The following traits were measured at harvest: plant height (i.e., the length of the plant from soil-line to the top of the main stem), number of scars (i.e., flower-abscission scars, representing failure to set seed), number of primary branches (i.e., flowering branches bifurcating from the main stem), number of secondary branches (i.e., flowering branches bifurcating from primary branches), number of main stem pods, and number of side branch pods (i.e., all pods produced in any plant part other than the main stem). After the above variables were measured, roots were separated and rinsed with water to remove adhering debris. Roots and above-ground plant parts were oven-dried at 80 °C for 48 h^95,96^, and weighed to estimate their biomass.

### Seed production and pollinator dependence

Pods of each focal plant were stored in separate paper bags and placed in a ventilated greenhouse to dry for a week. Pods were then oven-dried at 80 °C for 24 h before being threshed using a Haldrup LT-20 mechanical thresher and weighed to establish total seed biomass (i.e., seed production) in grams of seed per plant. The number of late maturing, and therefore un-matured, seeds per 1000 seeds (hereafter termed “green seeds”) in each plant were estimated with a Canola kit (Labtronics Canola Kit, Can-Seed Equipment Ltd), by counting distinctly green seed in 500 crushed main stem seeds and 500 crushed seeds from side branches.

We quantified pollinator dependence at the plant individual level using the RII index (Relative Inequality Index^97^), typically calculated as (X_pol_ − X_nopol_)/(X_pol_ + X_nopol_), where X_pol_ and X_nopol_ is the average total seed biomass in plants in the pollinator and no-pollinator treatments, respectively. We modified this formula to produce plant-level measurements, by comparing an individual plant’s total seed biomass to its varietal group mean in the other pollination treatment (either pollinator or no-pollinator). RII ranges from −1 to +1, is symmetrical around zero, has identical absolute values for dependence and non-dependence, and is continuous across its range^97^. Positive values indicate a higher seed yield in the presence of pollinators (or a higher pollinator dependence), while negative values indicate the opposite.

### Data analysis

All statistical analyses were performed using R version 3.4.2^98^. To evaluate the effect of the presence of pollinators, variety and variety type (OP vs H) on canola trait covariation, we first simplified our vegetative and phenological traits (see Supplementary Table S7) into four functional syndromes (flower timing, flowering effort, plant size and shape, seed packaging), and used principal components analysis (PCA; ‘vegan’ R package^99^) on each syndrome to produce 4 composite variables using the first principal component (PC1), each explaining 60–92% of the variation in the data (Supplementaty Table S8). We required that the variables summarized in each PC1 had correlations of >0.5 with the first PC eigenvector. For this reason, we isolated the trait “root biomass” as its own, non-PC functional trait. To meet the Pearson Correlation Coefficient assumption of bivariate normality, PCAs using correlations were run on variables to which we applied Box-Cox transformations whenever appropriate (Supplementary Table S7). We used the scores of these four 1^st^ principal component axes, along with root biomass as response variables in a multivariate analysis of variance (MANOVA) using the ‘manova’ function in the ‘stats’ package in R, with pollinator treatment (pollinators vs no-pollinators), variety type (OP vs H) and variety (23 levels, nested within variety type) as predictors, and all interactions included. Variety by pollination treatment pairwise comparisons were performed using the ‘pairwise.perm.manova’ function through the ‘RVAideMemoire’^100^ R package, applying an FDR correction for the number of contrasts^101^.

We tested for the effects of pollinators, variety type and variety nested within variety type, as well as of all measured vegetative and phenological traits, on total seed biomass (i.e., seed production), number of green seeds in 1000 seeds (i.e., “green seed”) and pollinator dependence, using generalized linear models. In the cases of total seed biomass and pollinator dependence, we used Gaussian (normal) errors with an identity link through the ‘glm’ function^98^, while for the green seed we used a negative binomial error distribution with a log link, using the ‘glm.nb’ function of the ‘MASS’ package^102^. We tested for the effects of presence of pollinators, variety type, variety (nested within variety type) and pollinator dependence on total seed biomass using generalized linear models (glm function) with Gaussian errors. We used variance inflation factors (VIF) to identify collinear explanatory variables that we removed from further analyses^103^. Collinearity was assessed with a cut-off value of 5^104^. In all cases, initial models included all ecologically meaningful interaction terms, and we simplified models by minimizing the Akaike Information Criterion (AIC), always retaining the non-significant main effects involved in higher order interactions, to satisfy the principle of marginality. In addition, initial models also tested for the random effect of block, but no significant spatial variation was revealed. Final models were checked for over-dispersion. When residuals of model fits were non-normal, we used the ‘boxCox’ function of the ‘car’ package^105^ to carry out Box-Cox transformations on non-normally distributed explanatory variables. To visualize our models, we used the ‘visreg’ function in the ‘VISREG’ package^106^ to extract adjusted data, visualized with partial residual plots using the ‘ggplot’ function of the ‘ggplot2’ package^107^.

### Statement of accordance

The authors declare that all the methods were carried out in accordance with the relevant guidelines and regulations.

## Supplementary information

Supplementary information

## Data Availability

The datasets analyzed during the current study are available from the corresponding author on reasonable request.
