# Supplementary material for: Pollinators enhance crop yield and shorten the growing season by modulating plant functional characteristics: A comparison of 23 canola varieties
Source: Sci Rep. 2019 Oct 2;9:14208. doi: 10.1038/s41598-019-50811-y (PMC6775066; doi:10.1038/s41598-019-50811-y)
Supplement: Supplementary file 1 — Supplementary information [file 41598_2019_50811_MOESM1_ESM.pdf]

Pollinators enhance crop yield and shorten the growing season by modulating plant functional characteristics: A comparison of 23 canola varieties

George C. Adamidis[1], Ralph V. Cartar[1], Andony P. Melathopoulos[2], Stephen F. Pernal[3], Shelley E. Hoover[4]

[1]Department of Biological Sciences, University of Calgary, Calgary, Canada
[2]Department of Horticulture, Oregon State University, Corvallis, OR, USA
[3]Agriculture and Agri-Food Canada, Beaverlodge Research Farm, Beaverlodge, AB, Canada
[4]Alberta Agriculture and Forestry, Lethbridge, AB, Canada

Keywords: crop pollination, oilseed rape, pollinator dependency

Table S1. Results of multivariate analysis of variance (MANOVA) for the effects of pollination, canola variety type, variety, and their interactions on flower timing, flower effort, plant size & shape, seed packaging and root biomass of canola plants grown under greenhouse conditions at the Lethbridge Research and Development Centre.

**Source of variation**	**Pillai's Trace**	***F***	**Df (num/den)**	***P***
Pollination	0.81	174.73	5/202	**<0.001**
Type	0.30	17.29	5/202	**<0.001**
Variety	1.33	3.57	105/1030	**<0.001**
Pollination x Type	0.04	1.74	5/202	0.12
Pollination x Variety	0.67	1.53	105/1030	**<0.001**

Table S2. Results of ANOVA (*F*-values and probabilities) for the univariate effects of pollination, canola variety type, variety, and their interactions on flower timing, flower effort, plant size & shape, seed packaging and root biomass of canola plants grown under greenhouse conditions at the Lethbridge Research and Development Centre. Differences between pollination treatments are identified.

**Source of variation**	***df***	**Flower timing**	**Flower effort**	**Plant size & shape**	**Seed packaging**	**Root biomass**
Pollination	1	39.62^{***}	382.45^{***}	24.71^{***}	12.79^{***}	35.94^{***}
Type	1	73.99^{***}	1.88	9.24^{**}	1.12	0.28
Variety	21	15.02^{***}	1.80^{*}	4.94^{***}	2.20^{**}	4.32^{***}
Pollination x Type	1	$3.10^{\cdot}$	$3.66^{\cdot}$	0.88	0.09	0.20
Pollination x Variety	21	1.71^{*}	2.43^{***}	1.75^{*}	0.88	1.42
Residual	206					
Differences				Pollinators < No-pollinators		

*******, P < 0.001; ******, P < 0.01; *****, P < 0.05

Table S3. Effects of pollination treatment, variety type, variety and vegetative and phenological traits and their interactions with pollination treatment on total seed biomass in 23 canola varieties grown under greenhouse conditions at the Lethbridge Research and Development Centre.

**Total Seed Biomass**				
**Coefficients**	**Estimate**	**Std. Error**	***t* value**	***P value***
Intercept	-10.82	1.183	-9.144	**<0.001**
Pollination	4.755	1.589	2.993	**0.003**
Variety type	-0.281	0.007	-3.988	**<0.001**
Variety	0.133	0.063	2.11	**0.036**
Peak of flowering	-927600	84640	-10.96	**<0.001**
Root biomass	0.467	0.077	6.063	**<0.001**
# Scars	0.020	0.073	0.281	0.779
Plant height	0.052	0.008	6.108	**<0.001**
#Primary branches	0.061	0.027	2.294	**0.023**
#Main stem pods	0.007	0.003	2.693	**0.008**
#Branch pods	0.037	0.025	1.478	0.141
Pollination x Peak of flowering	440300	128300	3.432	**<0.001**
Pollination x Plant height	-0.018	0.011	-1.62	0.106
Pollination x # Scars	-0.285	0.120	-2.366	**0.019**
Pollination x #Branch pods	0.268	0.090	2.984	**0.003**

*******, P < 0.001; ******, P < 0.01; *****, P < 0.05

Table S4. Effects of pollination treatment, variety type, variety and vegetative and phenological traits and their interactions with pollination treatment on the number of green seeds in 1000 seeds in 23 canola varieties grown under greenhouse conditions at the Lethbridge Research and Development Centre.

**Number of Green Seeds**				
Coefficients	Estimate	Std. Error	*z* value	*P* value
Intercept	-12.733	2.904	-4.384	**<0.001**
Pollination	7.884	4.372	1.803	0.071
Variety type	-0.088	0.223	-0.398	0.691
Variety	0.064	0.138	0.467	0.641
Peak of flowering	0.059	0.009	6.323	**<0.001**
#Flowers at peak	-0.025	0.009	-2.664	**0.008**
Root biomass	-1.208	0.584	-2.070	**0.038**
Plant height	-0.030	0.013	-2.380	**0.017**
#Primary branches	0.125	0.083	1.515	0.129
#Scars	-0.004	0.004	-1.111	0.266
Total #pods	0.003	0.002	1.255	0.209
Pollination x Variety type	0.852	0.312	2.733	**0.006**
Pollination x Peak of flowering	-0.026	0.014	-1.872	0.061
Pollination x Root biomass	1.352	0.650	2.080	**0.037**
Pollination x #Primary branches	-0.152	0.106	-1.440	0.149
Pollination x #Scars	0.008	0.004	1.759	0.078

*******, P < 0.001; ******, P < 0.01; *****, P < 0.05

Table S5. Effects of pollination treatment, variety type, variety and vegetative and phenological traits and their interactions with pollination treatment on pollinator dependence in 23 canola varieties grown under greenhouse conditions at the Lethbridge Research and Development Centre.

**Pollinator Dependence**				
Coefficients	Estimate	Std. Error	*t* value	*P* value
Intercept	-4.511	0.598	-7.539	**<0.001**
Pollination	8.904	0.819	10.872	**<0.001**
Variety type	-0.045	0.047	-0.961	0.337
Variety	-0.091	0.031	-2.908	**0.004**
Peak of flowering	-356000	43760	-8.135	**<0.001**
#Flowers at peak	0.204	0.066	3.114	**0.002**
Root biomass	0.100	0.050	2.017	**0.045**
Plant height	0.012	0.005	2.494	**0.013**
#Main stem pods	0.005	0.002	2.188	**0.030**
#Branch pods	0.023	0.013	1.780	0.076
Pollination x Variety type	-0.144	0.066	-2.162	**0.032**
Pollination x Peak of flowering	685900	64430	10.646	**<0.001**
Pollination x #Flowers at peak	-0.190	0.097	-1.951	0.052
Pollination x Root biomass	-0.308	0.077	-4.017	**<0.001**
Pollination x Plant height	-0.023	0.006	-3.700	**<0.001**
Pollination x #Main stem pods	-0.011	0.003	-3.852	**<0.001**
Pollination x #Branch pods	-0.112	0.045	-2.476	**0.014**

*******, P < 0.001; ******, P < 0.01; *****, P < 0.05

Table S6. Effects of pollination treatment, variety type, variety and pollinator dependence and their interactions on total seed biomass in 23 canola varieties grown under greenhouse conditions at the Lethbridge Research and Development Centre.

**Total Seed Biomass**				
**Coefficients**	**Estimate**	**Std. Error**	***t* value**	***P value***
Intercept	0.606	0.042	14.390	**< 0.001**
Pollinator Dependence	1.858	0.083	22.356	**< 0.001**
Pollination	0.539	0.048	11.160	**< 0.001**
Variety type	-0.373	0.048	-7.780	**< 0.001**
Variety	0.198	0.041	4.774	**< 0.001**
Pollinator Dependence x Variety type	0.309	0.102	3.032	**0.003**
Pollinator Dependence x Pollination	-4.565	0.104	-43.998	**< 0.001**

*******, P < 0.001; ******, P < 0.01; *****, P < 0.05

Table S7. Units and Box-Cox lambda exponents of the canola functional traits measured in this trial.

**Functional traits**	**Trait type**	**Units**	**Box-Cox Exponent (λ)**
Onset of flowering	Phenological	days	-2
Peak of flowering	Phenological	days	-2
Median of flowering	Phenological	days	-2
Cessation of flowering	Phenological	days	-1
Duration of flowering	Phenological	days	0
Number of flowers at the peak of flowering	Phenological	count	0
Total number of flowers	Phenological	count	0
Plant height	Vegetative	cm	1
Above-ground biomass	Vegetative	g	0
Root biomass	Vegetative	g	0
Number of scars	Vegetative	count	0
Number of primary branches	Vegetative	count	1
Number of secondary branches	Vegetative	count	0
Number of main stem pods	Vegetative	count	1
Number of branch pods	Vegetative	count	0
Total number of pods	Vegetative	count	0
Total seed biomass	Reproductive	g	0
Total number of green seeds in 1000 seeds	Reproductive	count	0

Table S8. Results of four principal components analyses on canola phenological and vegetative trait data, producing equal number of functional syndromes (i.e. flower timing, flowering effort, plant size and shape, seed packaging).

**Functional traits**	**1st Principal Component**	**Variation explained (%)**	**Trait loadings**
Number of flowers at the peak of flowering	Flowering effort	83.2	-0.93
Total number of flowers			-0.98
Duration of flowering			-0.82
Onset of flowering	Flower timing	91.6	-0.94
Peak of flowering			-0.96
Median of flowering			-0.99
Cessation of flowering			-0.94
Above-ground biomass	Plant size & shape	63.9	-0.92
Plant height			-0.84
Number of primary branches			-0.81
Number of secondary branches			-0.71
Number of scars	Seed packaging	60.0	-0.79
Number of main stem pods			-0.66
Number of branch pods			-0.87

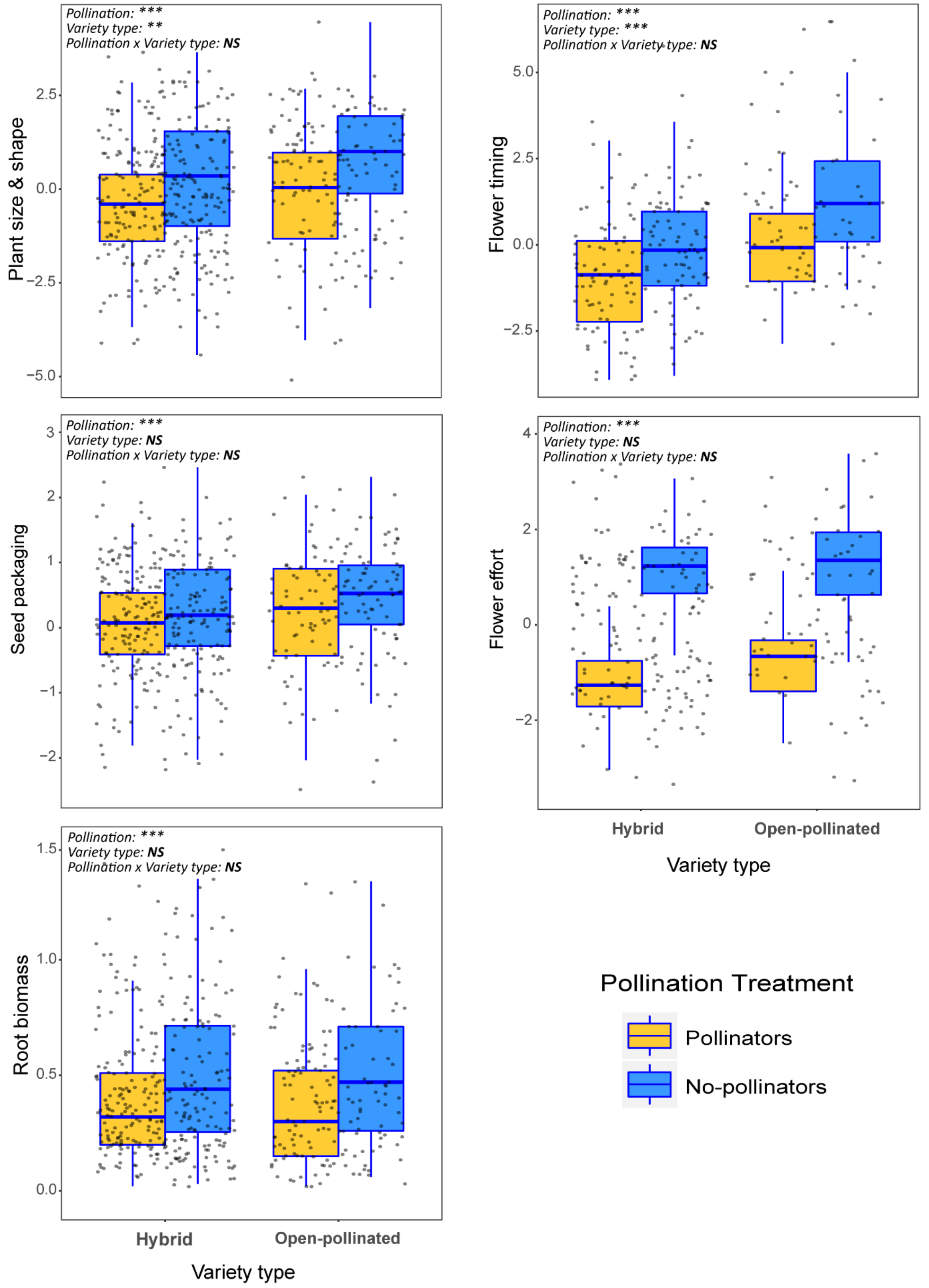

Figure S1. Box plots of the canola functional syndrome (see Table S2) and root biomass values in the presence and absence of bumble bees and in hybrid and open-pollinated canola varieties. The central horizontal line in the box plots represents the median of the samples, the box plot edges represent the first and third quartile. The interquartile range (IQR) within the boxes present the central 50% of the values. The whiskers show the range of observed values and the locations of the minimum and the maximum values. *******, $P < 0.001$; ******, $P < 0.01$; *****, $P < 0.05$; NS, not significant.

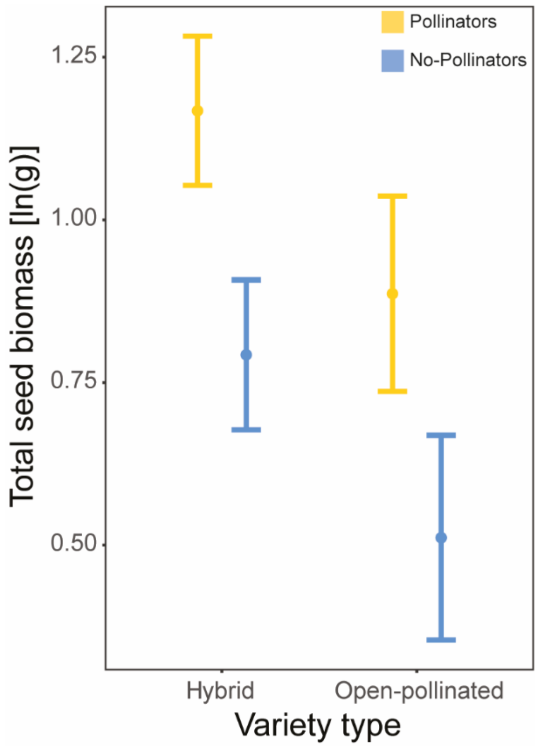

Figure S2. Model estimated mean total seed biomass production for hybrid and open-pollinated canola varieties in the presence (in yellow) and absence (in blue) of bumble bees. Error bars 95 % confidence intervals.

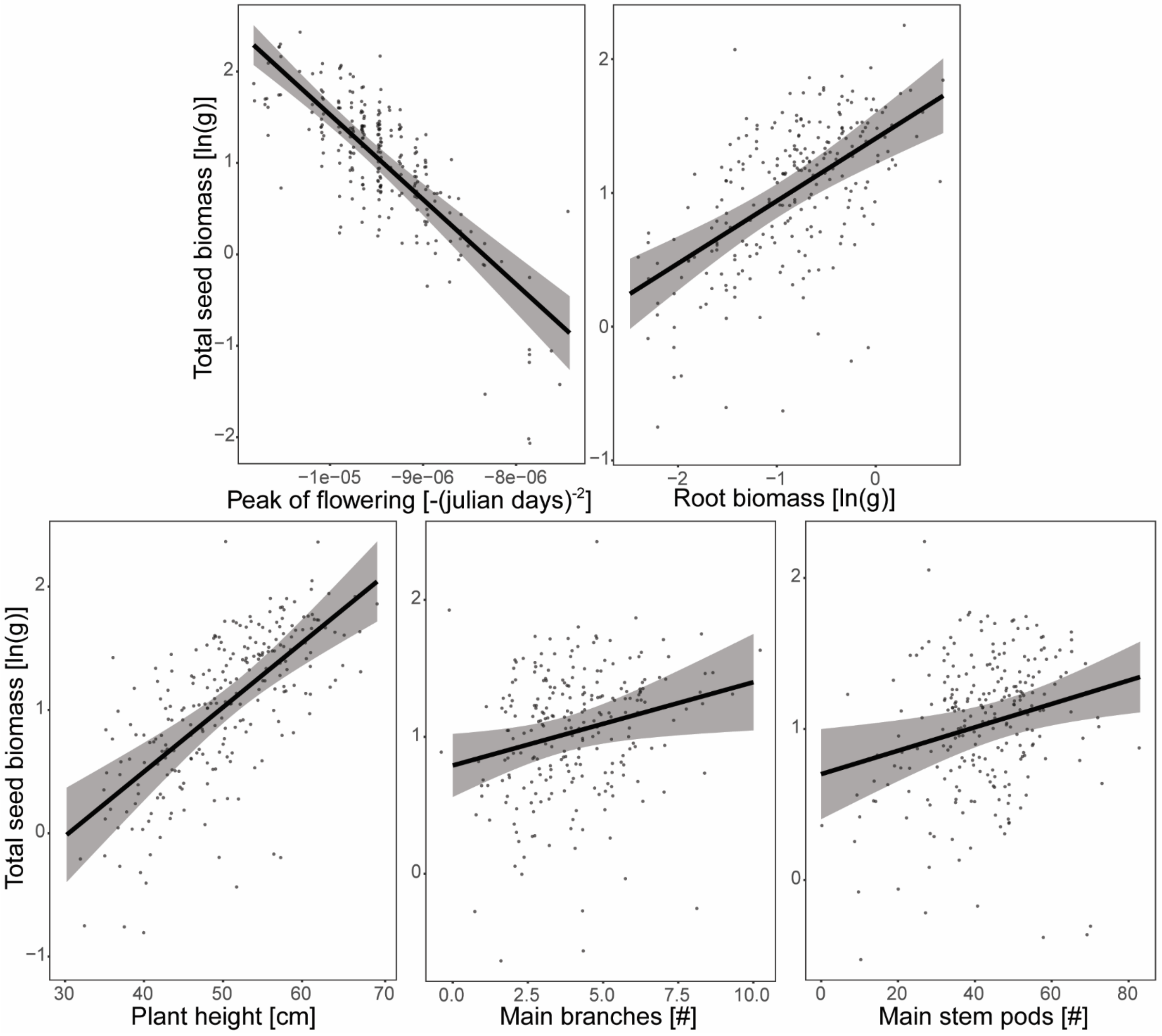

Figure S3. Partial residuals, prediction lines and confidence bands showing main effects of canola functional traits on total seed biomass.

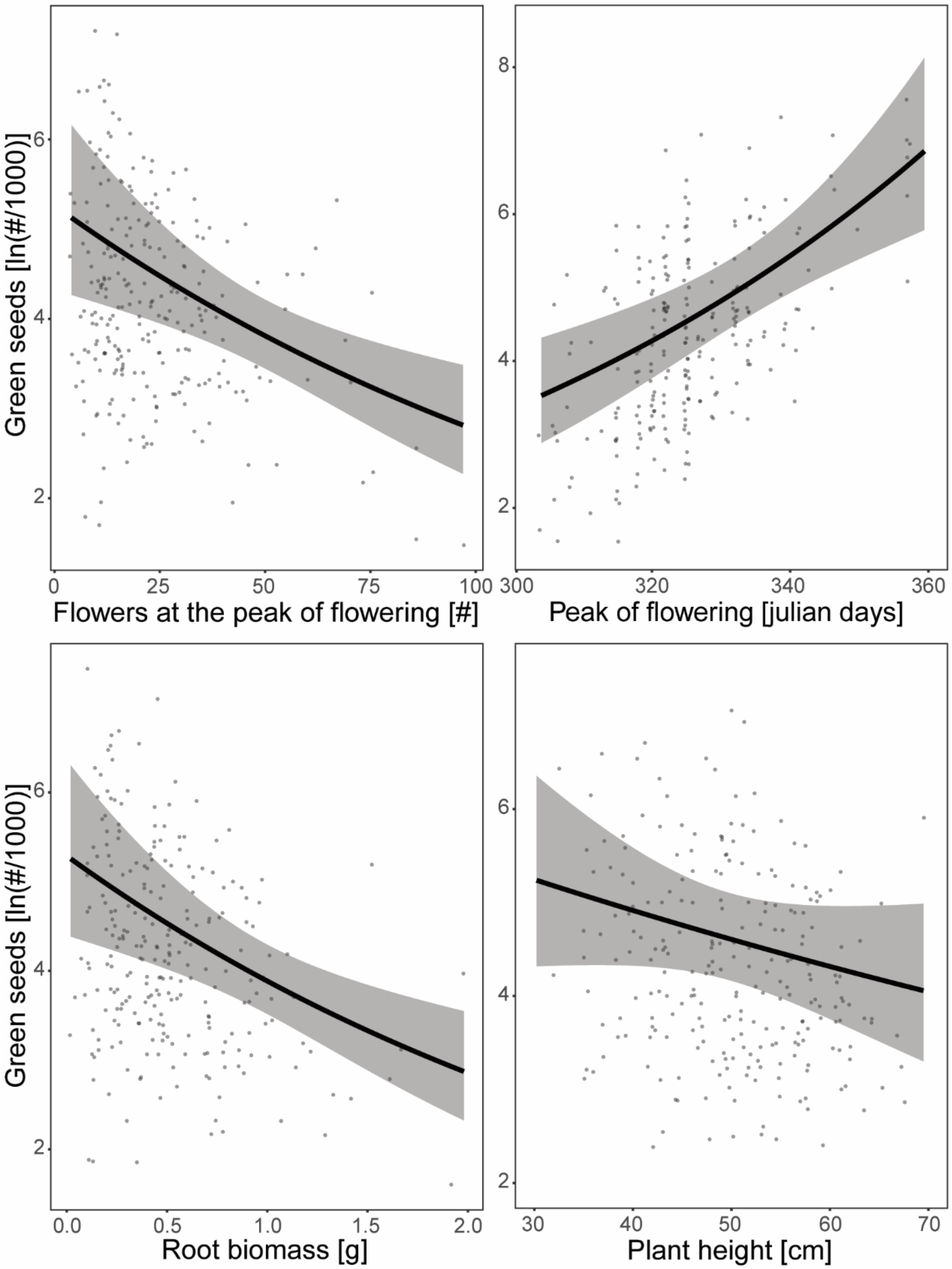

Figure S4. Partial residuals, prediction lines and confidence bands showing main effects of canola functional traits on number of green seeds per 1000 seeds.

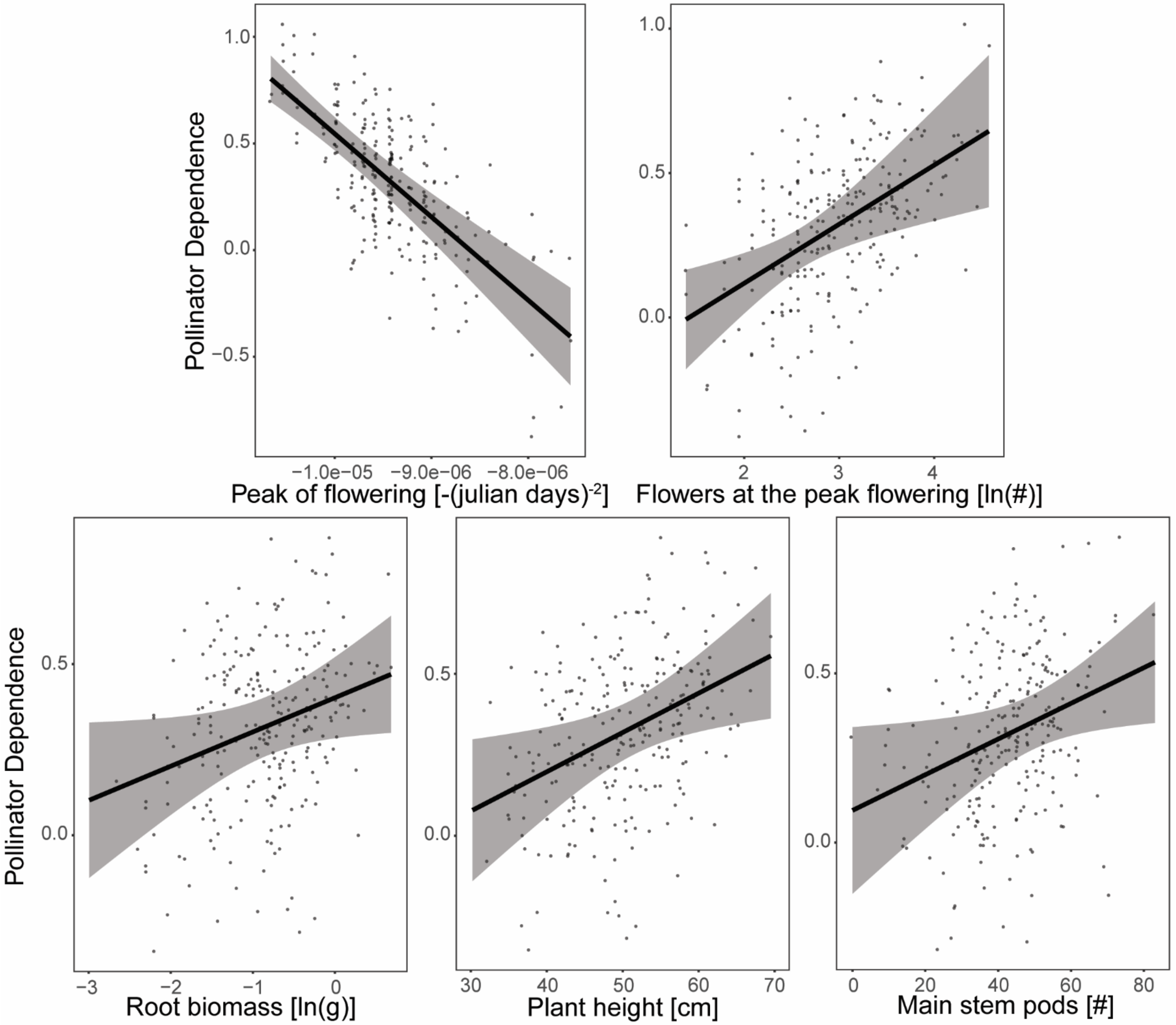

Figure S5. Partial residuals, prediction lines and confidence bands showing main effects of canola functional traits on pollinator dependence.

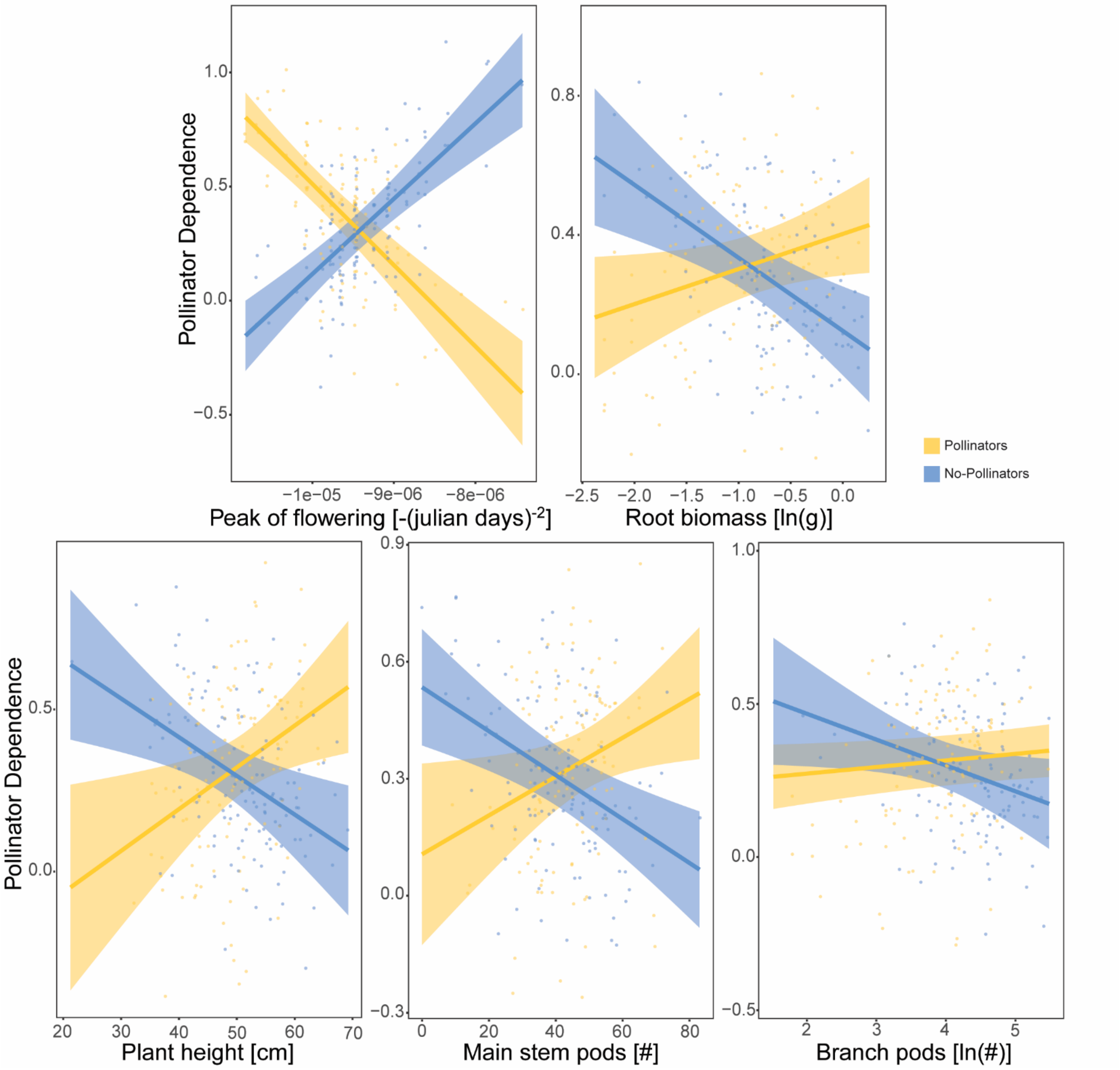

Figure S6. Partial residuals, prediction lines and confidence bands showing interactive effects of canola functional traits and pollination treatment (pollinators in yellow, no-pollinators in blue) on pollinator dependence.

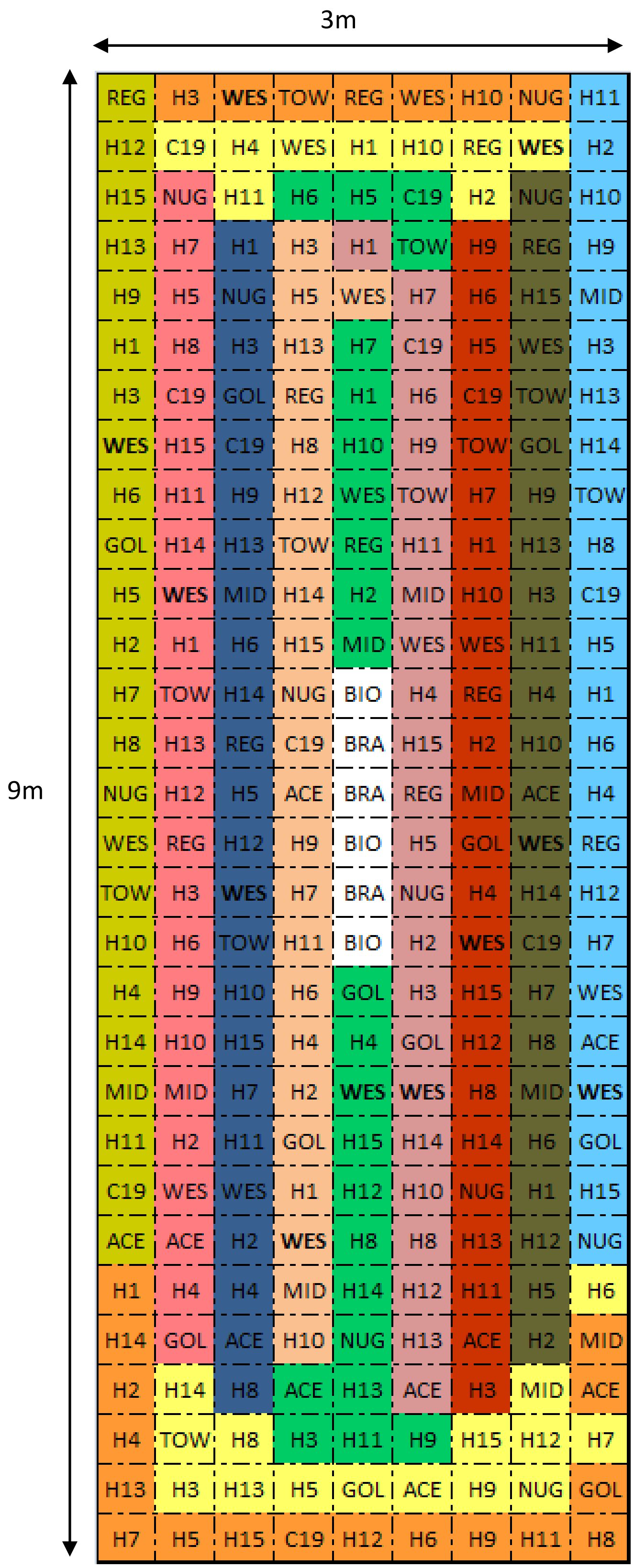

	Canola variety	Abbreviation
1	Hybrid variety 1	H1
2	Hybrid variety 2	H2
3	Hybrid variety 3	H3
4	Hybrid variety 4	H4
5	Hybrid variety 5	H5
6	Hybrid variety 6	H6
7	Hybrid variety 7	H7
8	Hybrid variety 8	H8
9	Hybrid variety 9	H9
10	Hybrid variety 10	H10
11	Hybrid variety 11	H11
12	Hybrid variety 12	H12
13	Hybrid variety 13	H13
14	Hybrid variety 14	H14
15	Hybrid variety 15	H15
16	Ac Excel	ACE
17	Canterra 1918	C19
18	Golden	GOL
19	Midas	MID
20	Nugget	NUG
21	Regent	REG
22	Tower	TOW
23	Westar	WES
	Westar covered with pollinator-excluding sleeve	**WES**
	Brassica juncea	BRA
	Capsicum spp. (biocontrol)	BIO

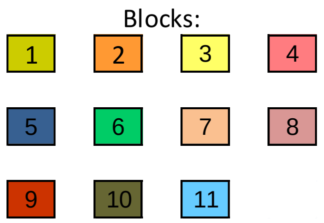

Figure S7. Canola varieties used in the trial and their arrangement and layout in the two greenhouses. Each cell represents a pot (270 pots in total in each greenhouse).
